# The Role of the Gut Microbiota and Uraemic Toxins in Vaccine Responsiveness Among People Receiving Maintenance Haemodialysis

**DOI:** 10.3390/vaccines14040358

**Published:** 2026-04-17

**Authors:** Erin Vaughan, Alexander Gilbert, Bree Shi, Griffith B. Perkins, Huiling Wu, Steve Chadban

**Affiliations:** 1 Renal Medicine, Kidney Centre, Royal Prince Alfred Hospital, Camperdown, NSW 2050, Australiasteve.chadban@health.nsw.gov.au (S.C.); 2 Faculty of Medicine and Health, University of Sydney, Camperdown, NSW 2050, Australia; 3 Central and Northern Adelaide Renal and Transplantation Service, Royal Adelaide Hospital, Adelaide, SA 5000, Australia; 4 School of Medicine, University of Adelaide, Adelaide, SA 5000, Australia

**Keywords:** chronic kidney disease, dialysis, vaccine responsiveness, gut microbiota, uraemic toxins, hepatitis B, SARS-CoV-2, indoxyl sulphate, p-cresyl sulphate, TMAO

## Abstract

**Background**: Patients with kidney failure requiring dialysis experience a high burden of vaccine-preventable diseases, and vaccine hypo-responsiveness is a key contributor. Uraemic toxins and gut dysbiosis are potential causes of hypo-responsiveness. **Aim**: This study aimed to determine whether uraemic toxin concentrations or gut dysbiosis are associated with vaccine response in haemodialysis patients. **Methods**: This was a single centre, observational cohort study of maintenance dialysis patients receiving a conventional 2-dose primary COVID-19 vaccination course. Demographic, clinical and vaccination data were collected from the eMR. Vaccine response (Elecsys Anti-SARS-CoV-2 immunoassay), serum uraemic toxin concentrations (indoxyl sulphate, p-cresyl sulphate, and trimethylamine N-oxide by liquid chromatography), and stool microbiome (16S rRNA gene sequencing) were measured 8 weeks after the second dose of vaccine. **Results**: Forty participants (43% female, mean age 66 years; 59% Caucasian) were included, 70% of whom were classified as a vaccine responder. Antibiotic exposure, prednisolone use and lymphopenia were significantly associated with hypo-responsiveness. Microbiome profiling identified differences in beta diversity between responders and non-responders, positively correlated with short-chain fatty acid producers (*Parabacteriodes*) and negatively with pathobionts (*Escherichia*/*Shigella*). Differential abundance analysis identified lower levels of *Tyzzerella*, *Gemmiger*, and *Hungatella* and higher levels of *Turicibacter* in vaccine responders. Total uraemic toxin burden and individual toxin concentrations did not differ between responders and hypo-responders (all *p* > 0.05). Stratification by low versus high/very high toxin burden groupings was not associated with response (*p* > 0.99). **Conclusions**: Differences in gut microbial composition were observed between vaccine responder groups, while uraemic toxin concentrations were not associated with vaccine responsiveness. These findings suggest gut microbiota composition may contribute to vaccine hypo-responsiveness in individuals receiving dialysis and warrant further investigation in larger mechanistic studies.

## 1. Introduction

Individuals with kidney failure receiving maintenance haemodialysis frequently exhibit an attenuated immune response to vaccination and remain at increased risk of vaccine-preventable infections [1,2,3]. Hypo-responsiveness to various vaccines has been documented, including hepatitis B (HBV) [4,5,6], influenza [7] and SARS-CoV-2 [8,9], and represents an area of unmet need among this vulnerable population. Traditional determinants, such as age, comorbidity, and malnutrition, contribute to impaired immunogenicity but do not fully explain the degree of hypo-responsiveness observed among dialysis patients [10,11].

Uraemia-induced immune dysregulation has been proposed as a possible mechanistic pathway. The chronic retention of protein-bound uremic toxins, in particular indoxyl sulphate (IS), p-cresyl sulphate (PCS), and trimethylamine N-oxide (TMAO), has been associated with impaired antigen presentation and inhibition of lymphocyte proliferation and is thought to promote systemic inflammation [11,12,13,14,15,16,17,18,19,20]. These toxins are poorly cleared by conventional dialysis, facilitating accumulation and downstream effects on immune function [21].

Dysbiosis of the gut microbiome is universal among the dialysis population. Whether this is a consequence of uraemia, dialysis dietary restrictions or other factors remains uncertain [22,23,24,25]. Haemodialysis patients exhibit reduced microbial diversity and enrichment of proteolytic taxa capable of generating uremic toxin precursors at the expense of relative depletion of bacteria capable of fermenting carbohydrate to generate short-chain fatty acids (SCFA) [26,27,28]. Such alterations may not only increase systemic toxin burden, but also disrupt mucosal barrier integrity and immune homeostasis through loss of SCFA-producing organisms and altered microbial signalling [29,30,31].

We hypothesised that dysbiosis-driven production of uraemic toxins represents a modifiable contributor to impaired vaccine responsiveness in haemodialysis. We conducted a cross-sectional association study within a longitudinally vaccinated cohort with the aim of investigating associations between uraemic toxin burden, gut microbial composition, and vaccine immunogenicity

## 2. Materials and Methods

### 2.1. Study Design and Population

Adult patients undergoing regular maintenance haemodialysis at a single facility were invited to participate in a cross-sectional cohort study to determine factors associated with their immune response to a standard 2-dose COVID-19 vaccine course. Inclusion criteria included agreement to receive 2 doses of a COVID-19 vaccine and willingness to provide samples of blood for vaccination response and uremic toxin measurement and stool for microbiota analysis. Patients with the following characteristics were excluded: prior COVID-19 infection, prior COVID-19 vaccination, vaccine allergy, hospitalisation within the month prior to study, active infection, or pregnancy. The study was approved by the University of Sydney ethics review board 2020/ETH01148, and all participants provided written informed consent.

**Figure 1:**
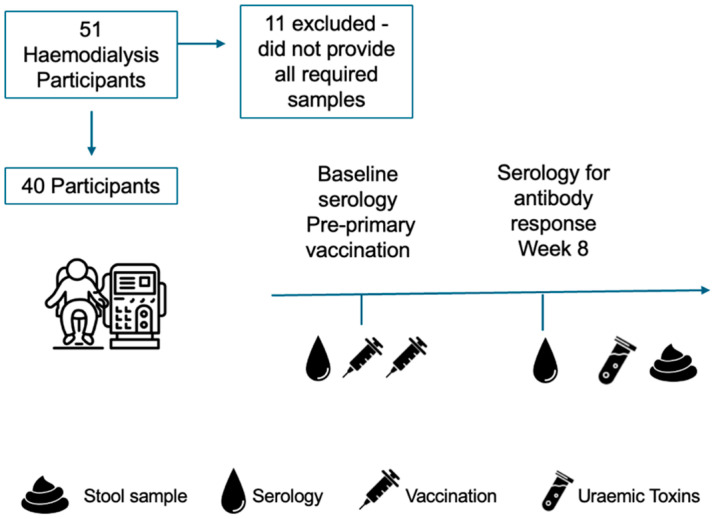
Study design.

Blood samples for serological analysis were collected at baseline (immediately prior to the first dose) and 8 weeks after completion of a 2-dose vaccination schedule. Serum uraemic toxin concentrations and faecal samples were collected 8 weeks after completion of the vaccination schedule (Figure 1).

Relevant clinical and demographic data were obtained from the electronic medical record for the study period. Information collected included baseline demographics, cause of kidney failure, dialysis access, comorbidities, vaccination status, and prescribed medications. Clinical outcomes recorded were hospital admissions (COVID-19, other infection, access-related, cardiac, or other), kidney transplantation, and death.

### 2.2. Serological Assessment of Vaccine Response

Humoral response to COVID-19 vaccination was assessed by quantifying anti-SARS-CoV-2 receptor-binding domain (RBD) and nucleocapsid (NC) antibodies using the Elecsys ‘Anti-SARS-CoV 2 S’ and ‘Elecsys Anti-SARS-CoV-2′ immunoassays, respectively, on the Cobas system (Roche, Basel, Switzerland) in accordance with the manufacturer’s instructions. The quantifiable range for the RBD-specific assay is 0.8–250 U/mL, with 1 U/mL equivalent to 1 binding antibody unit (BAU)/mL.

Vaccine responsiveness was defined using a dichotomized anti-RBD antibody concentration > 100 U/mL and anti-NC antibody negativity 8 weeks following completion of a two-dose vaccination schedule, to account for the known delayed immune response to vaccination in haemodialysis recipients and to exclude those who had experienced infection with SARS-CoV-2. Participants were classified as hypo-responders (anti-RBD < 100) or responders (anti-RBD > 100) at this time point. This categorization was used to facilitate clinically interpretable group comparisons of uraemic toxin burden, vaccine response, and microbiome profiles.

To minimise confounding from prior SARS-CoV-2 infection, participants with detectable anti-NC antibodies were excluded from analyses of vaccine-induced humoral response, irrespective of anti-RBD levels. Given the absence of a universally established protective anti-RBD threshold, antibody levels were secondarily analysed as a continuous variable.

### 2.3. Quantification of Uraemic Toxins

Serum concentrations of the protein-bound uraemic toxins indoxyl sulphate (IS) and p-cresyl sulphate (PCS) were quantified as both total and free fractions using ultrahigh-performance liquid chromatography (UPLC) with fluorescence detection. System control and data acquisition was performed using Empower3 chromatography data software (Waters Corporation, Milford, MA, USA). This method was validated by Pretorius et al. [32] and offers high sensitivity, with detection limits down to 0.1 µmol/L.

Plasma TMAO concentrations were measured by liquid chromatography–tandem mass spectrometry (LC–MS/MS) using an Acquity UPLC system with a Xevo TQD mass spectrometer (Waters, Milford, MA, USA). Data were processed with MassLynx software (version 4.2).

For analysis, 50 µL of plasma was deproteinised with 500 µL of acetonitrile containing a deuterated internal standard, followed by centrifugation for 10 min. A 1 µL aliquot of the supernatant was injected onto a BEH Amide column (1.8 µm, 2.1 × 100 mm; Waters). Separation was achieved using mobile phase A (100 mM ammonium formate, pH 3.0) and mobile phase B (acetonitrile), delivered at 0.4 mL/min. The column was maintained at 45 °C with a linear gradient from 0 to 25% A over 2.5 min, returning to baseline by 4 min. Quantification employed external calibration and an isotopically labelled internal standard. The calibration range was 0.1–200 µmol/L, with recovery between 98 and 102% and inter-day imprecision below 7% across four quality control levels.

### 2.4. Gut Microbiota Analysis

Stool samples were self-collected by participants using a standardised collection and microbial DNA stabilisation kit (OMNIgene GUT OM-200, DNA Genotek, Stittsville, ON, Canada). Samples were aliquoted and stored at −80 °C until further processing. Microbial DNA was extracted using the QIAamp DNA Stool Mini Kit (Qiagen, Hilden, Germany) according to the manufacturer’s protocol. Bacterial community profiling was performed by sequencing the V4 hypervariable region (515F-806R) of the 16S rRNA gene using paired high-end sequencing (2 × 250 bp) on the Illumina MiSeq platform at the Ramaciotti Centre for Genomics (University of New South Wales, Sydney, Australia).

Sequence data were processed using the DADA2 pipeline [33] implemented in R (version 4.4.2) within RStudio (2024.12.1 + 563), which included quality filtering, read merging, chimaera removal, and the generation of amplicon sequence variants (ASVs). Taxonomic assignment was conducted using the Ribosomal Database Project (RDP) naïve Bayesian classifier with species-level classification (rdp_train_set_18) [34]. ASVs representing < 0.01% of total reads were excluded from further analysis. Alpha diversity was evaluated using the Shannon index and observed richness, and beta diversity was assessed using Principal Coordinate Analysis (PCoA) Bray–Curtis dissimilarity matrices. Permutational multivariate analysis of variance (PERMANOVA) tests using the adonis function with 9999 permutations were conducted to assess for between-group differences, as well as to identify intrinsic and extrinsic factors explaining inter-individual variation in gut microbial composition. Differential abundance testing was performed using analysis of composition of microbiomes with bias correction of 2 (ANCOM-BC2) [35]. Spearman correlation was used to evaluate associations between bacterial genera with uraemic toxin levels and clinical parameters.

### 2.5. Statistical Analysis

Statistical analyses were undertaken in STATA (Version 18 SE; StataCorp, College Station, TX, USA). Continuous variables were assessed for normality and presented as mean ± SD or median (interquartile range IQR, Q1–Q3) as appropriate. Between-group comparisons were performed using independent-samples t-tests for normally distributed variables and Mann–Whitney U tests for non-normally distributed variables. Categorical variables were compared using Fisher’s exact test. All statistical tests were two-sided, with *p* < 0.05 considered statistically significant. Where appropriate, *p*-values were adjusted for multiple comparisons using the Benjamini–Hochberg false discovery rate (FDR) method.

Multivariable linear regression was used to examine the association between uraemic toxin burden and anti-RBD antibody levels. Models were adjusted for a limited number of clinically relevant covariates (prednisone or antibiotic use, lymphocyte count, and vaccine type) to minimise over-fitting, given the modest sample size. Robust standard errors were applied to account for potential deviations from model assumptions.

Vaccine response was primarily analysed as a dichotomous outcome (anti-RBD > 100 vs. <100 U/mL) for group-based comparisons. In addition, anti-RBD antibody levels were analysed as a continuous variable. Multivariable linear regression models were fitted with robust standard errors to account for heteroskedasticity. Sensitivity analyses were performed using log-transformed antibody titres.

Participants were grouped according to their toxin profiles using unsupervised clustering. K-means clustering (k = 3) was applied to classify individuals into subgroups with distinct toxin burdens. For visualisation, hierarchical clustering and heatmaps were generated using the ComplexHeatmap package in R (version 4.3.1; R Core Team, Vienna, Austria). Clustering was performed on scaled data using Euclidean distance and Ward’s minimum variance method (Ward.D). Extreme values were Winsorized at the 5th and 95th percentiles per toxin prior to scaling, to reduce the influence of outliers while retaining all observations. Heatmaps were colour-coded from low (blue) to high (red) to depict relative toxin abundance across clusters.

## 3. Results

### 3.1. Vaccine Response

Of 132 patients receiving maintenance dialysis at the facility, 51 participants met entry criteria and consented to the study, of whom 40 received both doses of vaccine, provided all required samples to assess SARS-CoV-2 vaccine response, and completed the study (Figure 1). Participant demographics are detailed in Table 1. Following a two-dose schedule with BNT162b2 (75%) or ChAdOx1 (25%) COVID-19 vaccines, 67% and 70% of recipients were classified as responders, respectively. The median age was comparable between vaccine response groups.

There was no difference in age, sex, ethnicity, or smoking status between vaccine responders and non-responders (Table 1). Non-responders were more likely to have glomerulonephritis as a cause of kidney failure, recent antibiotics, or current prednisolone therapy (*p* < 0.05).

### 3.2. Uraemic Toxin Concentration and COVID Vaccine Response

**Figure 2:**
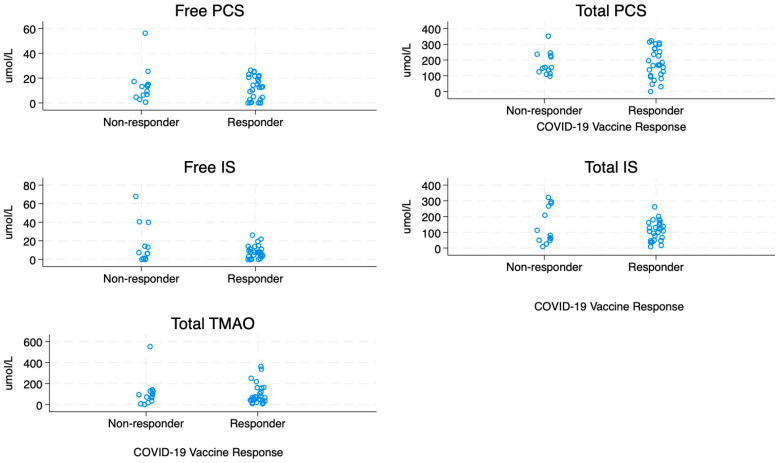
Uraemic toxin concentrations according to vaccine response status. Uraemic toxin concentrations are shown for participants stratified by responder status. Differences between responders and non-responders are illustrated for each measured toxin. Values represent individual measurements with group-level distributions displayed.

Uraemic toxin concentrations were measured and compared according to responder status (Figure 2). Free and bound fractions of IS, PCS, and TMAO were higher than reference values reported for healthy individuals but did not differ by vaccine-response category (Table 2). The median total toxin burden was also similar between responders (386 (228–468) µmol/L) and non-responders (360 (260–526) µmol/L) (*p* = 0.74).

**Figure 3:**
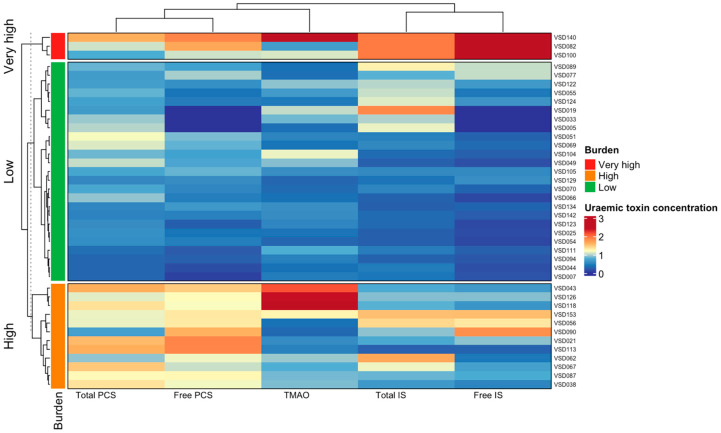
Clustering of participants based on toxin profiles. Heatmap showing relative toxin abundance across participants following unsupervised clustering. Individuals were grouped into three clusters representing distinct toxin burden profiles. Colours indicate scaled toxin levels ranging from low (blue) to high (red). Rows represent individual participants and columns represent toxins.

To further characterise toxin exposure, K-means clustering based on scaled uraemic toxin concentrations identified three distinct burden groups: very high (n = 3, 7.5%), high (n = 12, 30%), and low (n = 25, 62.5%) burden (Figure 3). While individual toxin medians varied across clusters, the very-high burden group demonstrated the greatest overall toxin load (Appendix A). Vaccine response rates were similar between the High/Very high toxin burden group and the low burden group, with no statistically significant association between toxin burden and vaccine responder status (*p* > 0.99).

In a secondary exploratory multivariable analysis, composite toxin burden was not significantly associated with anti-RBD titres after adjustment for clinical covariates (all *p* > 0.05), although an inverse association was observed in sensitivity analyses using log-transformed titres (*p* < 0.001).

### 3.3. Uraemic Toxin Burden and Clinical Outcomes

No statistically significant associations were observed between toxicity group and adverse clinical outcomes. COVID-19 infection not requiring hospital admission occurred in 67% of participants with high/very high toxin burden and 36% of those with low toxin burden (*p* = 0.10). Hospital admission rates were 40% in the high/very high group and 68% in the low group (*p* = 0.11). There was no significant difference in hospital admission for COVID-19 infection between high and low toxin burden groups (*p* = 0.38).

### 3.4. Biochemical and Clinical Associations with Vaccine Response Status

Baseline laboratory parameters were evaluated for associations with SARS-CoV-2 vaccine responsiveness. Responders (n = 27) had significantly higher mean lymphocyte counts compared with non-responders (1.54 × 10^9^/L (± 0.75) vs. 1.04 × 10^9^/L (± 0.48); *p* = 0.03). Mean albumin (34.8 g/L vs. 33.5 g/L) and haemoglobin levels (110 g/L vs. 105 g/L) were not different between responders and non-responders (*p* = 0.25 and *p* = 0.29, respectively). Pre-dialysis urea levels were similar between groups (21.2 mmol/L vs. 18.9 mmol/L; *p* = 0.20).

No significant differences in adverse outcomes were observed. Hospital admissions (77% vs. 48%; *p* = 0.10), rates of non-admission COVID-19 infection (54% vs. 44%; *p* = 0.74), other illnesses (46% vs. 37%; *p* = 0.73), and mortality (8% vs. 7%; *p* = 1.00) were comparable between responders and non-responders, respectively.

Among participants with confirmed COVID-19 infection (n = 24), six (25%) required hospitalisation—three non-responders (33%) and three responders (20%) (*p* = 0.64). COVID-19 severity did not differ significantly between groups (1.0 vs. 1.33, *p* = 0.06).

### 3.5. Gut Microbiota Composition and Vaccine Responses

**Figure 4:**
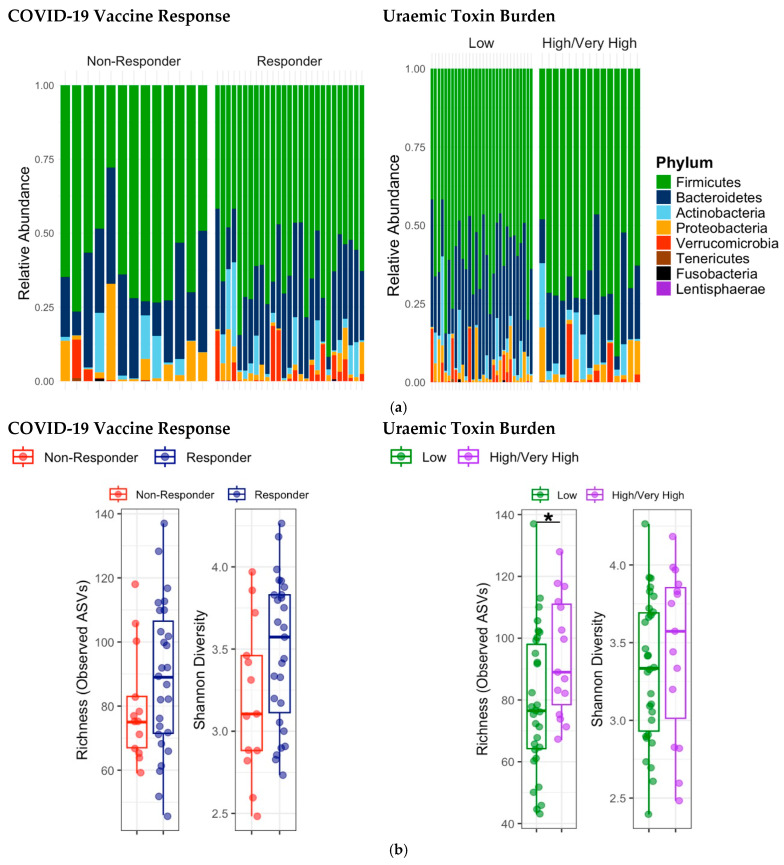

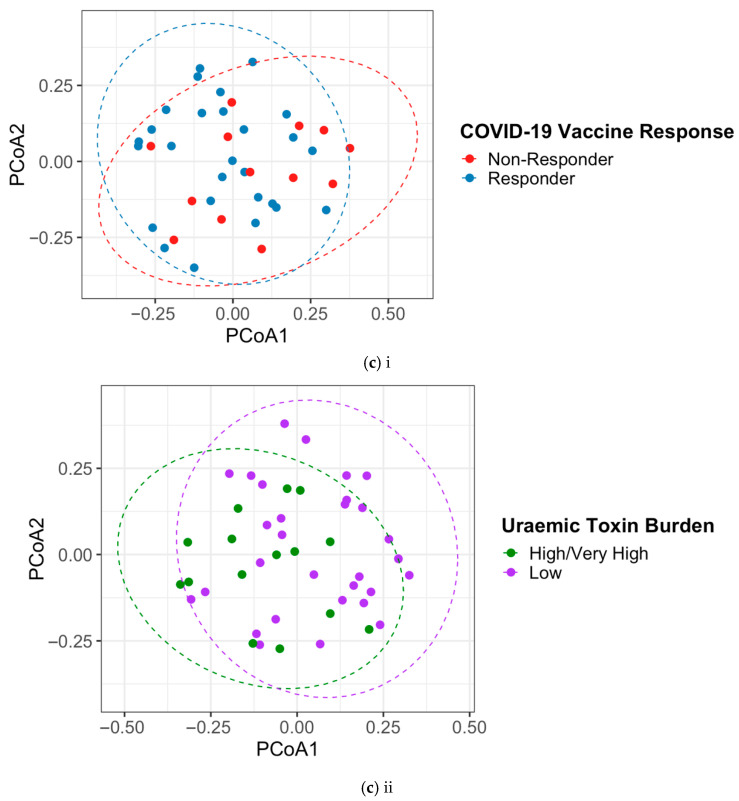
(**a**) Relative abundance of gut microbial taxa according to responder status and circulating uraemic toxin concentrations. (**b**) Alpha diversity between vaccine responders and uraemic toxin burden, * *p* < 0.05. (**c**) Beta diversity by (**i**) vaccine responder status and (**ii**) uraemic toxin burden.

At the phylum level, no significant differences in relative abundance were observed between SARS-CoV-2 vaccine responder groups (Figure 4a). Alpha diversity did not differ significantly between SARS-CoV-2 vaccine responder groups (observed richness: *p* = 0.24; Shannon index: *p* = 0.09, Figure 4b). Beta diversity, assessed by Principal Coordinate Analysis (PCoA) of Bray–Curtis distances, demonstrated a significant difference in overall microbial community composition between vaccine responders and non-responders (R^2^ = 0.04, *p* = 0.03, PERMANOVA). PCoA plots were consistent with these findings, showing partial clustering by vaccine response (Figure 4c(i)).

**Figure 5:**
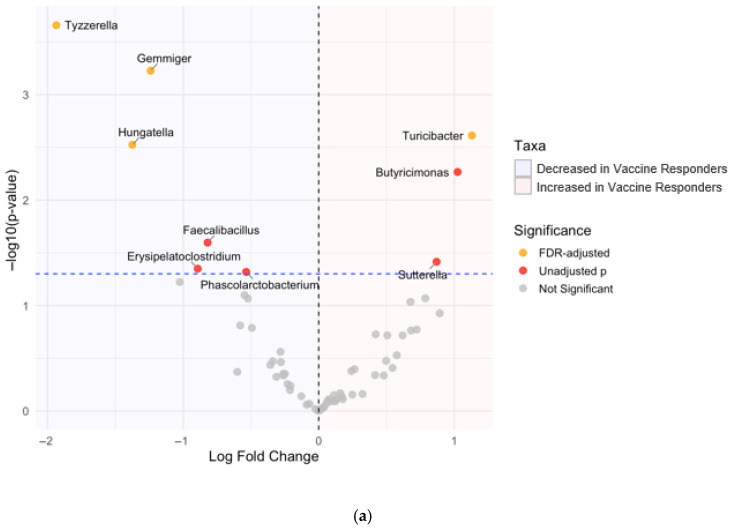

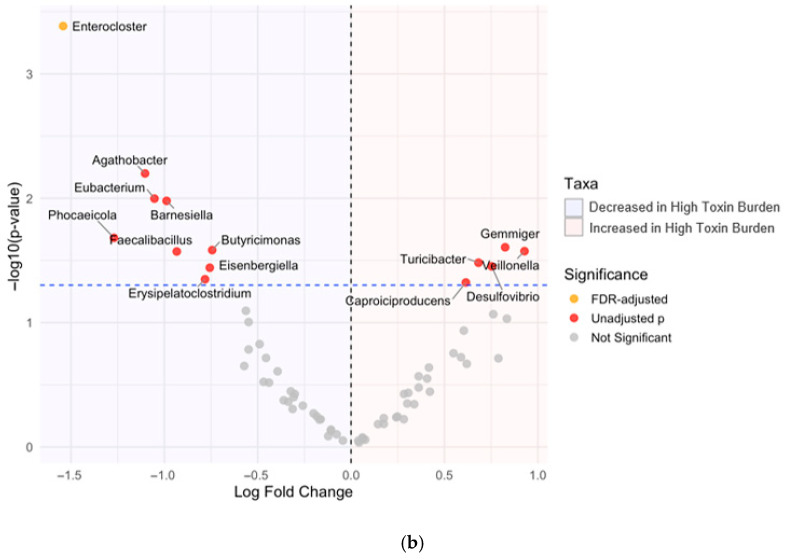
Differential abundance of gut microbial taxa between (**a**) vaccine responder status and (**b**) uraemic toxin burden. Labelled taxa above the horizontal dashed line achieved unadjusted *p*-values < 0.05, and taxa in yellow remained significant after false discovery rate correction (FDR-adjusted *p*-value < 0.05).

Differential abundance testing using ANCOM-BC2 identified several genera associated with vaccine response status (Figure 5). Vaccine responders exhibited a significantly lower relative abundance of *Gemmiger*, a taxon not previously associated with dysbiosis; *Tyzzerella* and *Hungatella*, which have been associated with dysbiosis; systemic inflammation; and uraemic toxin production. Conversely, responders showed an increased relative abundance of *Turicibacter*, a genus potentially implicated in immune activation, after false discovery rate (FDR) adjustment.

### 3.6. Gut Microbiota and Uraemic Toxin Concentrations

Increased abundance of Actinobacteria (log fold change 1.21, unadjusted *p* = 0.04) and Verrucomicrobia phyla (log fold change 1.12, unadjusted *p* = 0.05) were evident in participants with high uraemic toxin burden, compared to low burden (Figure 4a). Higher observed richness was found among patients with high toxin burden compared to those with low toxin burden (mean 94.4 vs. 79.2, *p* = 0.03), while Shannon diversity remained comparable (*p* = 0.36, Figure 4b).

Beta diversity demonstrated no significant separation between participants with low and high uraemic toxin burden (*p* = 0.24). PCoA plots were consistent with these findings, with substantial overlap between toxin burden groups (Figure 4c(ii)).

Differential abundance testing using ANCOM-BC2 was stratified by uraemic toxin burden. The high-toxin group showed a significant FDR-adjusted decrease in Enterocloster, while the unadjusted analyses indicated relative increases in Gemmiger, Turicibacter, Veillonella, Caproiciproducens, and Desulfovibrio (Figure 5). PERMANOVA models based on Bray–Curtis distances were performed to identify clinical and biochemical factors that were associated with the most explained variance (R^2^) in gut microbial composition between individuals. Among these, significant associations were observed with uraemic toxins: free indoxyl sulphate (R^2^ = 0.03, *p* = 0.03), total p-cresyl sulphate (R^2^ = 0.03, *p* = 0.03), and total indoxyl sulphate (R^2^ = 0.03, *p* = 0.04).

**Figure 6:**
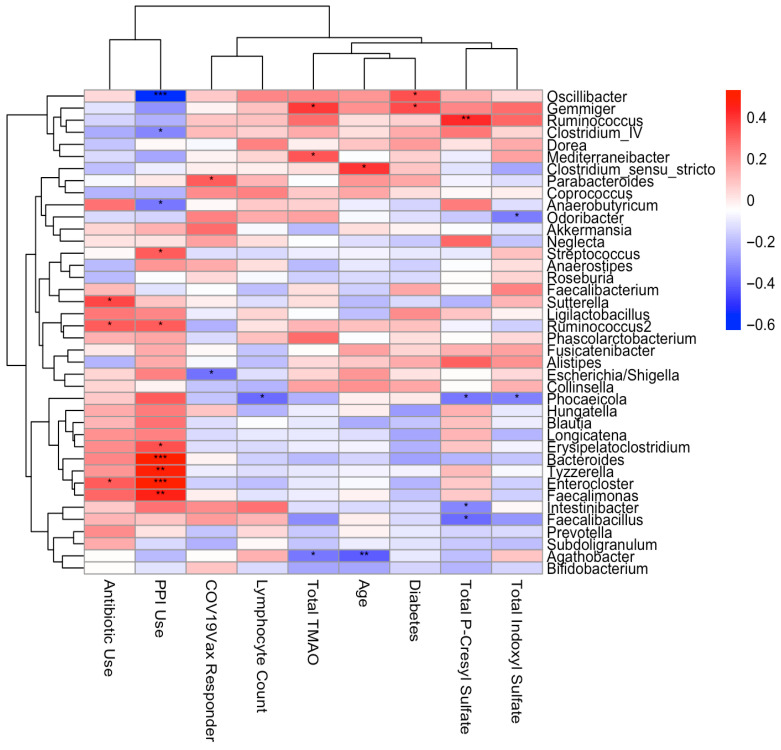
Spearman correlation heatmap of top 40 most abundant genera and associations with clinical and biochemical variables. The strength and direction of correlations varied across taxa, with significance thresholds denoted as *p* < 0.05 (*), *p* < 0.01 (**), and *p* < 0.001 (***).

Spearman correlation analysis found significant associations between uraemic toxin concentrations and specific bacterial genera (Figure 6). Total TMAO concentrations correlated positively with Gemmiger and Mediterraneibacter, and negatively with Agathobacter. Total PCS concentration correlated negatively with Faecalibacillus, Intestinibacter, and Phocaeicola, but positively with Ruminococcus 2. Total IS concentrations were negatively correlated with Phocaeicola and Odoribacter (Figure 6).

### 3.7. Gut Microbiota and Clinical Associations

At the genus level, Spearman correlation analysis demonstrated that antibiotic exposure was associated with increased relative abundance of Enterocloster, Ruminococcus 2, and Sutterella, whereas lymphocyte count was inversely associated with Phocaeicola (Figure 6).

**Figure 7:**
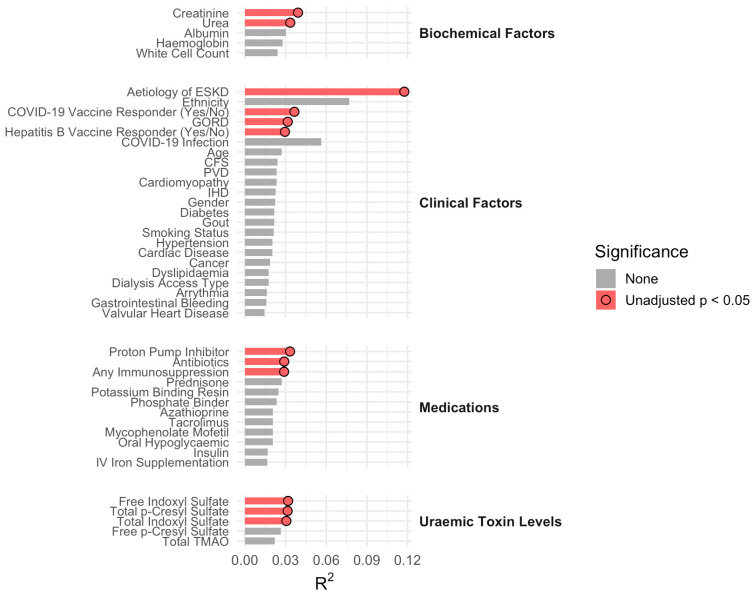
PERMANOVA explained variance (Bray–Curtis distance). Biochemical, clinical, medication and uraemic toxin variables associated with inter-individual variation in gut microbial composition. Bars represent the proportion of variance (R^2^) explained by each variable, and red bars denote those significantly associated with beta diversity (*p* < 0.05).

PERMANOVA based on Bray–Curtis dissimilarities identified several clinical and biochemical variables associated with inter-individual variation in gut microbial composition. The cause of kidney failure was the dominant contributor, explaining 12% of the total variance in microbial community structure (R^2^ = 0.12). Additional covariates contributing to microbial variation included serum creatinine, urea concentration, vaccine responder status, and exposure to proton pump inhibitors, immunosuppressive therapies, and antibiotics (Figure 7).

## 4. Discussion

To our knowledge, this is the first study to explore integrated relationships between gut microbiota composition, circulating uraemic toxin concentrations, and vaccine responsiveness in maintenance haemodialysis recipients. Uremic toxin accumulation has previously been implicated in immune dysfunction in dialysis recipients [17]. We found that circulating uraemic toxin concentrations were elevated, consistent with kidney failure, but did not differ between vaccine responders and non-responders when defined using a dichotomised antibody threshold. Circulating concentrations of IS, PCS, and TMAO did not distinguish responders from non-responders, nor were concentrations of uremic toxins associated with clinical events. Our primary findings therefore fail to support any relationship between uremic toxin burden and COVID-19 vaccine hypo-responsiveness. To explore the risk that arbitrary dichotomization based on toxin burden may obscure more subtle relationships, we conducted an exploratory analysis modelling toxin concentration as a continuous variable with adjustment for clinical determinants of vaccine response. No significant association with antibody response was observed; however, sensitivity analyses using log-transformed antibody titres suggested a potential association between higher burden and reduced responsiveness. Thus, whilst our primary data findings suggest that uremic toxins are unlikely to be important determinants of vaccine response in dialysis patients, we must wait for larger studies to confirm this hypothesis.

### 4.1. Vaccine Response and Gut Microbiome

The potential role of the gut microbiome in shaping vaccine responses has been increasingly recognised, although underlying mechanisms remain incompletely understood [37,38,39,40]. Prior studies in non-CKD cohorts have demonstrated associations between gut microbial composition and SARS-CoV-2 vaccine immunogenicity [41,42,43]. In the present study, differences in overall microbial composition were observed between responders and non-responders; however, the effect size was modest.

At the genus level, differences in relative abundance were observed between groups. However, these findings should be interpreted with caution. Our study utilised 16S rRNA sequencing, which provides genus-level resolution but does not allow direct inference of microbial function or metabolic activity. Furthermore, the modest sample size and potential sources of variability limit the ability to draw firm conclusions regarding specific taxa.

Several of the genera identified in this study have been associated with immune and metabolic processes in other contexts. For example, *Parabacteriodes* and *Turicibacter* have been linked to host–microbial signalling and epithelial barrier function [44,45], whereas *Escherichia/Shigella*, *Tyzzerella*, and *Hungatella* have been associated with inflammatory states and proteolytic metabolism [46,47,48]. Although functional interpretation is limited, as genus-level classification does not allow direct assessment of microbial function, these findings are biologically plausible and broadly consistent with patterns reported in the literature [48].

An important limitation is that in our study, microbiome and metabolite sampling occurred after vaccination. It remains unclear whether the observed microbial differences preceded vaccine responsiveness or were influenced by intervening factors such as medication exposure, illness, or the vaccine itself. Collectively, the limitations of sample timing and 16S sequencing methodology enable us to support the hypothesis that perturbations in the gut microbiome may influence vaccine responsiveness in dialysis, but do not provide proof of this concept.

### 4.2. Uraemic Toxins and Microbiota

Uraemic toxin production is closely linked to microbial function in CKD cohorts [49,50,51]. CKD is associated with depletion of carbohydrate-fermenting commensals and expansion of proteolytic taxa, a shift linked to circulating toxin accumulation [52,53], systemic inflammation [54,55], and adverse kidney outcomes [25,56]. These observations support the concept of a metabolically ‘toxic’ microbiome in advanced kidney disease. In keeping with this, higher toxin burden was associated with differences in microbial diversity, including increased alpha diversity. This finding may reflect the expansion of metabolically unfavourable taxa, rather than the restoration of a balanced microbial ecosystem, as has been observed in other dialysis cohorts [57]. Beta diversity analyses did not demonstrate clear separation by uraemic toxin burden, suggesting that uraemic toxin accumulation may relate more to functional or metabolic shifts within communities than to overall community composition.

Manipulation of the gut microbiota to decrease uraemic toxins remains an active area of investigation. Microbiome-targeted interventions and dietary modification have been shown to lower circulating uraemic toxins (PCS and IS) in CKD [58,59], highlighting the gut–kidney axis as a potentially modifiable pathway. In this context, our findings suggest that microbial community structure may represent more than a marker of uraemic toxin burden and instead form part of a broader framework linking dysbiosis, uraemic metabolism, and immune response.

### 4.3. Clinical Impacts

Variation in gut microbial composition was also associated with several clinical and demographic factors, highlighting the multifactorial influences shaping the gut microbiome in this population. Antibiotic exposure and prednisone use were associated with reduced vaccine responsiveness, consistent with their known effects on immune function and the gut microbiome [60,61,62,63]. Together, these associations raise the possibility that microbiome alterations may contribute to variability in vaccine response, although causality cannot be established from the current data.

Vaccine platform may also influence immune responses through differences in antigen delivery and immune activation. In this cohort, vaccine type was not independently associated with antibody response; however, given the modest sample size, we cannot exclude a potential influence of vaccine platform on microbiome composition or vaccine responsiveness.

The observed seroconversion rates following SARS-CoV-2 vaccination of ~70% in this study (Table A1) mirror those reported in comparable cohorts [64,65,66], and highlight impaired immunity compared with healthy cohorts in our previous studies, who consistently achieved seropositivity rates (anti-RBD Ig > 100 U/mL) of 100% following two SARS-CoV-2 vaccine doses [67,68]. We found that vaccine responders demonstrated higher lymphocyte counts, suggesting relative preservation of immune competence compared with non-responders. Despite this, rates of COVID-19 infection, hospitalisation, and mortality were similar between groups, indicating that serological response alone did not translate into detectable differences in short-term clinical outcomes. This aligns with evidence that vaccine protection in kidney failure reflects contributions from both humoral and cellular immunity, and that antibody titres alone incompletely capture protective immunity [3]. It is possible that cellular immune responses may remain relatively intact and continue to limit disease severity, even in the absence of robust serological responses, potentially accounting for the absence of observable differences in clinical outcomes.

Strengths of this study include its integrative design, combining microbiome sequencing, uraemic toxin profiling, and vaccine serology within a single, well-characterised cohort. The use of multiple complementary analytic approaches, including analyses of microbial diversity, clustering methods, and differential abundance testing, adds robustness to the interpretation. Limitations include the relatively small cohort size, single-centre design, and cross-sectional sampling, which preclude causal inference. The absence of dietary data and cellular immune profiling further limits interpretation. Accordingly, all findings should be considered exploratory and hypothesis-generating.

These findings suggest that vaccine hypo-responsiveness in dialysis is likely multifactorial. Uraemic toxin concentrations were not associated with vaccine response when analysed using dichotomized thresholds, although exploratory continuous analyses suggest a potential graded relationship with antibody concentrations. Differences in gut microbial composition were observed between responder groups, but these accounted for only a small proportion of overall variation, and functional interpretation remains limited. Overall, these findings support a potential role for the microbiome in contributing to variability in vaccine responsiveness. Further longitudinal studies incorporating pre-vaccination sampling and functional microbiome analyses are needed to clarify these relationships and inform strategies to improve vaccine response in this high-risk population.

## Figures and Tables

**Table 1:** Baseline characteristics by SARS-CoV-2 vaccine response status.

	Responder N = 27 n (%)	Non-Responder N = 13 n (%)	*p* Value
**Age (median, IQR)**	65 (26–87)	66 (48–81)	0.94
**Male**	15 (56)	8 (62)	0.72
**Race/ethnicity**			0.51
**Aboriginal**	6 (22)	1 (8)	
**Black**	1 (4)	0	
**Caucasian/white**	13 (48)	10 (77)	
**Asian**	3 (11)	1 (8)	
**North African/Middle Eastern**	4 (15)	1 (8)	
**Smoker**	3 (12)	1 (8)	0.65
**Cause of kidney failure**			0.04
**Diabetes**	11 (41)	5 (38)	1.00
**Hypertension/vascular**	5 (19)	0	0.15
**Polycystic kidney disease**	1 (4)	2 (15)	0.24
**Glomerulonephritis**	2 (7)	5 (38)	0.03
**Other ^**	2 (7)	1 (8)	1.00
**Unknown**	6 (22)	0	0.08
**Vascular access**			0.31
**AV fistula**	20 (74)	8 (62)	
**AV graft**	0	1 (8)	
**Vascular catheter**	7 (26)	4 (31)	
**Vaccine**			0.85
**Pfizer**	20 (74)	10 (77)	
**AZ**	7 (26)	3 (23)	
**Comorbidity**			
**Diabetes**	13 (48)	6 (46)	0.91
**Hypertension**	19 (70)	8 (62)	0.58
**Peripheral Vascular Disease**	6 (22)	2 (15)	0.61
**Cardiac disease**	14 (52)	8 (62)	0.56
**Ischaemic heart disease**	9 (33)	5 (38)	0.75
**Valvular heart disease**	0	0	
**Cardiomyopathy**	1 (4)	1 (8)	0.59
**Cancer**	3 (11)	1 (8)	0.74
**Gout**	2 (7)	4 (31)	0.05
**Gastrointestinal bleeding**	3 (11)	0	0.21
**Gastrointestinal reflux disease**	3 (11)	2 (15)	0.70
**Dyslipidaemia**	5 (19)	6 (46)	0.07
**Medications**			
**Antibiotics**	3 (11)	5 (38)	0.04
**Phosphate binders**	17 (63)	4 (31)	0.06
**Potassium-binding resin**	3 (11)	3 (23)	0.32
**Iron (intravenous)**	18 (67)	7 (54)	0.43
**Erythropoietin**	22 (81)	12 (92)	0.37
**Proton pump inhibitor**	10 (37)	5 (38)	0.93
ARB/ACEi **	6 (22)	2 (15)	0.61
**Statin**	10 (37)	8 (62)	0.14
**Calcitriol**	12 (44)	4 (31)	0.41
**Caltrate**	10 (37)	4 (31)	0.70
**Insulin**	4 (15)	4 (31)	0.24
**Aspirin**	9 (33)	8 (62)	0.09
**Cinacalcet**	4 (15)	3 (23)	0.52
**Immunosuppression**			
**Prednisone**	1 (4)	5(38)	0.004
**Azathioprine**	0	1 (8)	0.14

^ Other: Lithium toxicity, myeloma, scleroderma, multifactorial (calculi, hypertension); ** ARB, angiotensin receptor blocker, ACEi, angiotensin converting enzyme inhibitor.

**Table 2:** Uraemic toxin concentrations and total toxin burden by vaccine response.

Toxin	Reference Range (μmol/L) [32,36]	Responders (n = 27)	Non-Responders (n = 13)	*p*-Value
Free PCS (µmol/L), median (IQR)	0.14–2.44	10.3 (3.9–17.6)	10.6 (7.0–16.6)	0.68
Total PCS (µmol/L) ^1^, mean (±SD)	0.0–38.4	175.7 ± 99.5	182.8 ± 77.0	0.91
Free IS (µmol/L), median (IQR)	0.0–0.19	7.4 (3.2–12.7)	4.1 (2.1–25.1)	0.77
Total IS (µmol/L), median (IQR)	0.70–6.30	109 (58–147)	72 (48–262)	0.78
Total TMAO (µmol/L), median (IQR)	1.28–19.67	67 (46–104)	81 (45–111)	0.85
Total toxin burden (µmol/L), median (IQR)		386 (228–468)	360 (260–526)	0.74

^1^ Normally distributed; data are presented as mean ± SD and compared using an independent-samples *t*-test. All other variables are presented as median (interquartile range, Q1–Q3) and compared using the Mann–Whitney U test.

## Data Availability

The data presented in this study are not publicly available due to privacy and ethical restrictions related to the protection of participant confidentiality. Individual-level data cannot be shared as this would compromise participant privacy in accordance with the conditions of the ethics approval granted by the University of Sydney Human Research Ethics Committee. Aggregated data supporting the findings of this study may be made available from the corresponding author upon reasonable request and subject to approval by the relevant ethics committee.

## Abbreviations

The following abbreviations are used in this manuscript:

ACEi	Angiotensin-converting enzyme inhibitor
ANACOM-BC2	Analysis of composition of microbiomes with bias correction of 2
BAU	Binding antibody unit
CKD	Chronic kidney disease
HBV	Hepatitis B vaccine
IS	Indoxyl sulphate
LC-MS	Liquid chromatography–tandem mass spectrometry
NC	Nucleocapsid
PCoA	Principal Coordinate Analysis
PCS	P-Cresyl sulphate
PERMANOVA	Permutational multivariate analysis of variance
RBD	Receptor-Binding Domain
SFCA	Short-chain fatty acids
TMAO	Trimethylamine N-oxide

## Appendix A

**Table A1:** Two-way frequency table of COVID-19 and hepatitis B responders.

	SARS-CoV-2 Responder	SARS-CoV-2 Non-Responder	Total	*p*-Value
**Hepatitis B Vaccine**				0.25
**Responder**	16 (76)	5 (24)	21	
**Non-responder**	6 (55)	5 (45)	11	
**Total**	22	10	32	

**Table A2:** Median uraemic toxin concentration by cluster.

Uraemic Toxin Cluster	Free pCS	Total pCS	Free IS	Total IS	Total TMAO
**Low**	6.7	136	3.9	64	57
**High**	18.4	253.5	11.2	121.5	103
**Very high**	24	209	41	297	130
